# Healthcare Expenditures among US Immigrants with Cognitive Limitations or Alzheimer’s Disease and Related Dementias

**DOI:** 10.1002/alz.088801

**Published:** 2025-01-09

**Authors:** Seyeon Jang, Xuanzi Qin, Sungchul Park, Rozalina G. McCoy, Jie Chen

**Affiliations:** ^1^ University of Maryland School of Public Health, College Park, MD USA; ^2^ The Hospital And Public health interdisciplinary research (HAPPY) Lab, School of Public Health, University of Maryland, College Park, MD USA; ^3^ Korea University, Seoul Korea, Republic of (South); ^4^ L‐HOPE Program for Community‐Based Total Learning Health Systems, Korea University, Seoul Korea, Republic of (South); ^5^ University of Maryland School of Medicine, Baltimore, MD USA; ^6^ University of Maryland Institute for Health Computing, Bethesda, MD USA

## Abstract

**Background:**

Despite the growing number and proportion of the immigrant population in the US, there is a lack of knowledge on healthcare expenditures among immigrants with cognitive limitations or Alzheimer’s disease and related dementias (ADRD). This study estimated differences in total healthcare expenditures between US‐born and foreign‐born individuals by the presence of ADRD and/or cognitive limitations. This study also examined the differences in healthcare expenditures by service types, including inpatient stays, office‐based visits, emergency department (ED) visits, home healthcare, and prescription drugs.

**Method:**

The study used nationally representative data from the 2007‐2020 Medical Expenditure Panel Survey (MEPS). Generalized linear regression models were used to estimate differences in healthcare expenditures between US‐born and foreign‐born individuals with ADRD, cognitive limitations without ADRD, and without ADRD or cognitive limitations. Analyses were also implemented by the immigrants’ duration of residence in the US, classified as foreign‐born living less than 10 years in the US and more than 10 years in the US. The study sample included all community‐dwelling adult Americans and had separate subpopulation analyses on the elderly population aged 65 and above. Survey weights were applied to all estimates.

**Result:**

Foreign‐born individuals with cognitive limitations or ADRD had substantially higher total healthcare expenditures than their counterparts. Compared to US‐born individuals with ADRD, foreign‐born individuals with ADRD spent significantly more on home healthcare ($4,456.1, *p* = 0.029). Among individuals without ADRD or cognitive limitations, foreign‐born individuals spent significantly less than their US‐born counterparts in most healthcare services. Compared to their US‐born counterparts, immigrants with ADRD who had lived in the US for more than 10 years encountered significantly higher home healthcare costs ($4,245.6, *p* = 0.031), whereas those who had lived in the US for less than 10 years incurred significantly lower costs on office‐based visits (‐$1,833.8, p<0.001) and ED visits (‐$514.4, p<0.001). In contrast, immigrants with no ADRD and cognitive limitations spent significantly less than their US‐born counterparts on healthcare regardless of residence duration in the US.

**Conclusion:**

Foreign‐born individuals with cognitive limitations or ADRD had substantially higher total healthcare expenditures than their US‐born counterparts, while foreign‐born individuals without ADRD or cognitive limitations spent significantly less than their US‐born counterparts.

